# Supplementary material for: A High-Throughput Screening System Based on Droplet Microfluidics for Glucose Oxidase Gene Libraries
Source: Molecules. 2020 May 22;25(10):2418. doi: 10.3390/molecules25102418 (PMC7287683; doi:10.3390/molecules25102418)

Supplementary material

A high-throughput screening system based on droplet microfluidics for glucose oxidase gene libraries

Radivoje Prodanović [1,2,*], W. Lloyd Ung [2], Karla Ilić Đurđić [1], Rainer Fischer [3], David A. Weitz [2] and Raluca Ostafe [4]

[1] Faculty of Chemistry, University of Belgrade, Studentski trg 12, 11000 Belgrade, Serbia; karlailic@chem.bg.ac.rs

[2] Department of Physics, School of Engineering and Applied Sciences, Harvard University, Cambridge, MA 02138, USA; lloyd.ung@gmail.com (W.L.U.); weitz@seas.harvard.edu (D. A.W.)

[3] Departments of Biological Sciences and Chemistry, Purdue University, 207 S. Martin Jischke Dr., West Lafayette, IN 47907, USA; fische70@purdue

[4] Purdue Institute of Inflammation, Immunology and Infectious Disease, Molecular Evolution, Protein Engineering and Production, Purdue University, 207 S. Martin Jischke Dr., West Lafayette, IN 47907, USA; rostafe@purdue.edu

* Correspondence: rprodano@chem.bg.ac.rs; Tel.: +38-111-333-6660

Table 1. Primers for consensus library.

1	R37K	GACTCACCACCGCTGCTAAACTGACGGAGAACCCCAAC
2	S53F	TCATCGAAAGTGGCTTTTACGAGTCGGACAGAGG
3	V106I	CTCGGTGGCTCTACTCTAATTAATGGTGGCACCTGGAC
4	A192T	GGCTCTCATGAGCACTGTCGAAGACCG
5	N278D	CACAAGGGCAACACCCACGATGTTTACGCTAAGCACGAG
6	V293I	CCGCGGGCTCCGCTATTTCTCCCACAATCCTC
7	E310D	GAAGTCCATCCTGGATCCCCTTGGTATCGAC
8	E374D	CAACACCAAGCTGGATCAGTGGGCCGAAG
9	I403L	GAACTACCGCGACTGGTTGGTCAACCACAACGTCG
10	L429I	CTTCGATGTGTGGGACCTTATTCCCTTCACCCGAGGATAC
11	I474L	GCTACTCAACTGGCCCGCAACTTGTCCAACTCCGG
12	H510N	GACTGAGTACATCCCGTACAATTTCCGTCCTAACTACCATG
13	M528L	CATGATGCCGAAGGAGTTGGGCGGTGTTG
14	R537K	GTGTTGTTGATAATGCTGCCAAAGTGTATGGTGTGCAGGGAC
15	M556V	GTTCTATTCCTCCTACGCAAGTTTCGTCCCATGTCATGACGG
16	I597V	CGCCTGGACTGAGTACGTTCCGTACCACTTCCGTC

Co-flow encapsulation device
25 um channels

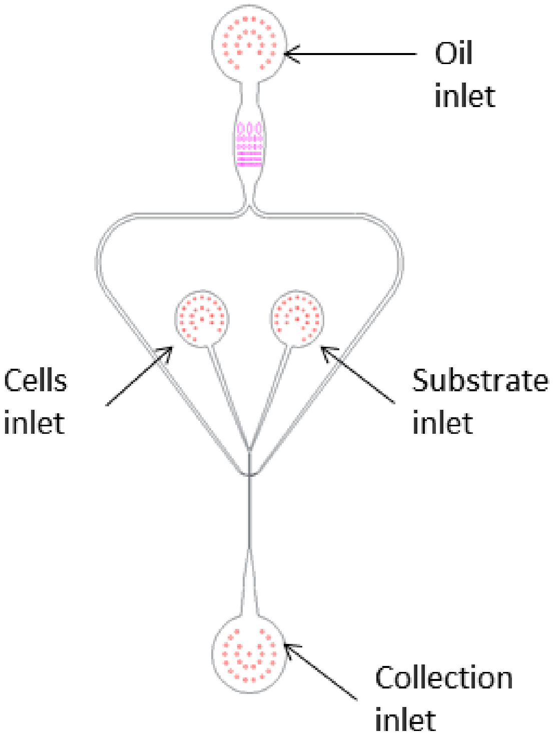

Sorting device 25 um channels

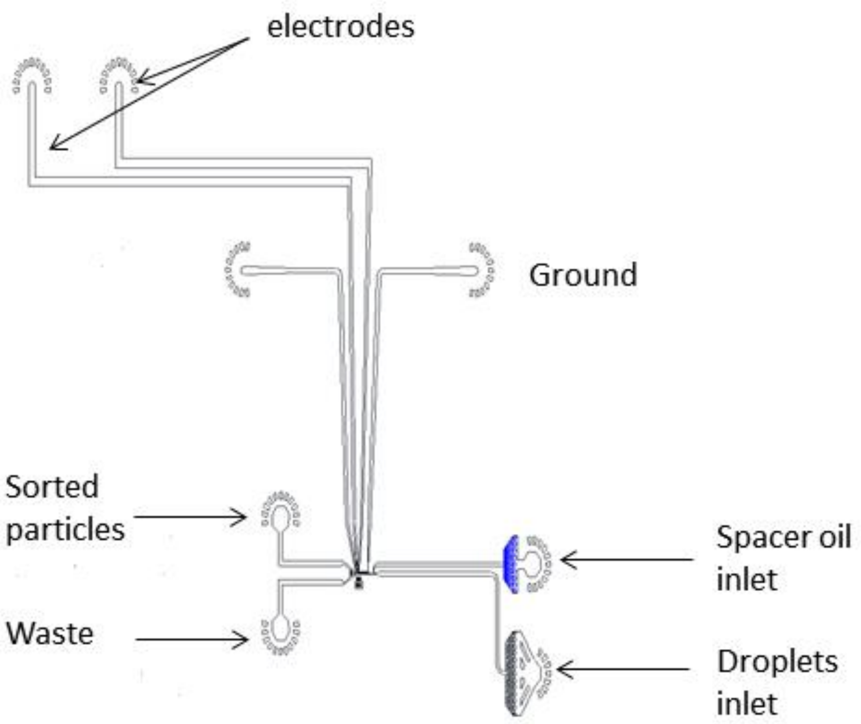

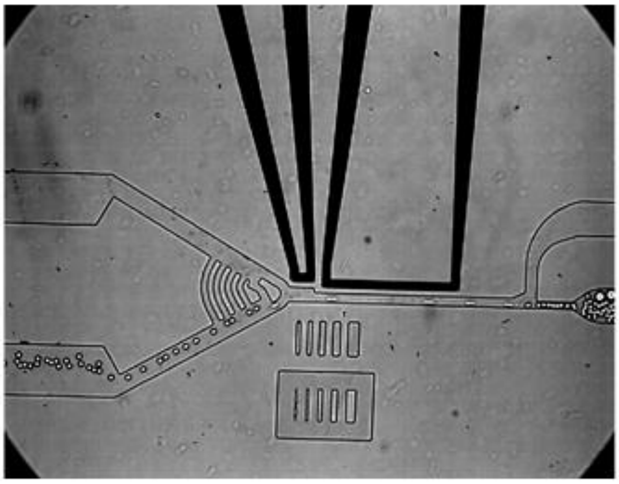

Supplement: Supplementary file 1 [file molecules-25-02418-s001.pdf]
